# Perception of the impact of oral issues on the quality of life of Indigenous populations of Espírito Santo, Brazil

**DOI:** 10.1590/0102-311XEN159825

**Published:** 2026-05-29

**Authors:** Deise Berger Velten, José Geraldo Mill, Lorrayne Cesario Maria, Maria Helena Monteiro de Barros Miotto

**Affiliations:** 1 Universidade Federal do Espírito Santo, Vitória, Brasil.

**Keywords:** Quality of Life, Oral Health, Indigenous Population, Qualidade de Vida, Saúde Bucal, Povos Indígenas, Calidad de Vida, Salud Bucal, Pueblos Indígenas

## Abstract

The disparities in oral health between Indigenous and non-Indigenous people can be seen as unfair. Although researchers have documented the precarious oral health of Indigenous people, some authors argue that there should be better research. This study evaluated the impact of oral issues on the quality of life and the association of sociodemographic characteristics, use of dental services, and need for removable dentures in the Indigenous population of the municipality of Aracruz, Espírito Santo State, Brazil. In this cross-sectional study, the perceived quality of life of 1,084 Guarani and Tupiniquim individuals was evaluated by the *Oral Health Impact Profile* (OHIP). Other variables were assessed by an adapted questionnaire. The associations between each independent variable and OHIP dimensions were assessed using the chi-squared and Fischer’s exact tests. The strength of these associations was assessed by odds ratio. This study found that 495 Indigenous individuals reported an impact on their quality of life (a 45.7% prevalence). Physical pain and psychological discomfort had the highest percentages of impact. Individuals aged up to 50 years, with a self-reported need for removable partial dentures, and without a need for removable total dentures reported a greater perception of the impact of oral issues on their quality of life. The prevalence of impact of oral issues on quality of life of Indigenous individuals exceeded that of non-Indigenous ones in Aracruz, which shows this population’s vulnerable oral health.

## Abstract (pt)

As disparidades em saúde bucal entre indígenas e não-indígenas podem ser vistas como injustas. Embora pesquisadores tenham documentado a precariedade da saúde bucal dos povos indígenas, alguns autores argumentam em favor de uma investigação melhor. Este estudo avaliou o impacto dos problemas orais na qualidade de vida e sua associação de características sociodemográficas, uso de serviços odontológicos e necessidade de dentaduras removíveis na população indígena do Município de Aracruz, Espírito Santo, Brasil. Um estudo transversal foi conduzido no qual 1.084 membros guarani e tupiniquim foram avaliados em relação à sua percepção da qualidade de vida usando o questionário *Oral Health Impact Profile* (OHIP; Perfil de Impacto em Saúde Bucal). Outras variáveis foram avaliadas por meio de outro questionário adaptado. Utilizou-se os testes qui-quadrado e exato de Fischer para verificar as associações entre cada variável independente e as dimensões do OHIP. Razões de chances foram usadas para avaliar a força das associações encontradas. A prevalência do impacto geral foi de 45,7%, com 495 indígenas relatando impacto na sua qualidade de vida. “Dor física” e “desconforto psicológico” tiveram as maiores porcentagens de impacto. Indivíduos com até 50 anos de idade, com necessidade autodeclarada de próteses parciais removíveis e sem necessidade de dentaduras totais removíveis relataram uma percepção maior do impacto dos problemas orais na sua qualidade de vida. A prevalência do impacto das questões orais na qualidade de vida em aldeias indígenas do Município de Aracruz foi maior do que entre indivíduos não indígenas, o que demonstra a vulnerabilidade dessa população na saúde bucal.

## Abstract (es)

Las disparidades encontradas en la salud bucodental entre la población indígena y la no indígena pueden considerarse injustas. Aunque los investigadores han documentado la precariedad de la salud bucodental de los indígenas, algunos autores sostienen que debería investigarse más a fondo. Este estudio evaluó el impacto de los problemas bucodentales en la calidad de vida y la asociación de las características sociodemográficas, el uso de los servicios dentales y la necesidad de prótesis removibles en la población indígena del municipio de Aracruz, Espírito Santo, Brasil. Se trata de un estudio transversal en el que se evaluó a 1.084 miembros de los pueblos guaraní y tupiniquim en relación con la percepción de la calidad de vida mediante el cuestionario *Oral Health Impact Profile* (OHIP; Perfil del Impacto en la Salud Bucal), y se evaluaron otras variables mediante otro cuestionario adaptado. Se utilizaron las pruebas de chi-cuadrado y exacta de Fischer para verificar las asociaciones entre cada variable independiente y las dimensiones del OHIP, y la *odds ratio* para evaluar la fuerza de la asociación. La prevalencia del impacto general fue del 45,7%, con 495 indígenas que informaron del impacto en la calidad de vida. Las dimensiones “dolor físico” y “malestar psicológico” tuvieron los porcentajes más altos de impacto. Las personas de hasta 50 años, que declararon necesitar prótesis parciales removibles y que no necesitaban prótesis totales removibles, informaron una mayor percepción del impacto de los problemas bucales en su calidad de vida. La prevalencia del impacto de los problemas bucales en la calidad de vida de los pueblos indígenas del municipio de Aracruz fue mayor que la de las personas no indígenas, lo que demuestra la vulnerabilidad de esta población en materia de salud bucal.

## Introduction

Oral health is an essential aspect of well-being as a whole; as a part of general health, it interferes in quality of life and thus exceeds the idea that it would be otherwise limited to tooth care. Normative indicators are unable to capture problems such as pain, interference in mastication, and self-esteem; these aspects are instead measured by questionnaires that evaluate the impact of oral issues on quality of life. Thus, to find the impact of oral diseases on people’s daily lives, researchers started discussing the concept of “quality of life” regarding oral health ^1^.

The way in which oral health influences quality of life has been widely investigated using indices that evaluate individuals’ functional and social perceptions, enabling a better understanding of feelings, self-perceived oral health and expectations regarding the treatment of populations ^2^.

Quality of life has been thought of in terms of the degree of satisfaction in family, love, social, and environmental life and to existential aesthetics, and can be defined as an eminently human notion. It describes the ability to culturally synthesize all the elements a given society considers its standard of comfort and well-being. Quality of life encompasses many meanings that, in turn, reflect the knowledge, experiences, and values of individuals and collectivity ^3^. A variety of conditions that affect individuals’ perception, senses, and behaviors during their daily activities determine oral health-related quality of life; hence the growing interest in quantifying the consequences of a disease that affects its carriers’ routine ^4^.

The *Oral Health Impact Profile* (OHIP) was developed in Australia to identify the impact oral problems have on quality of life. Its first version had 49 questions ^5^. Then, three years after its creation, Slade developed a shortened version of the OHIP questionnaire and reported initial findings regarding reliability and validity, thus obtaining the OHIP-14, which retains the original conceptual dimensions in the OHIP and can thus detect associations between social impact and perceived need for treatment at increasing hierarchical levels of impact. The questions are to be answered in a Likert scale. They encompass functional limitation, physical pain, psychological discomfort, physical disability, psychological disability, social disability, and handicap. According to its authors, the OHIP-14 can detect previously observed associations between social impact and perceived need for treatment ^5^ ^,^ ^6^. Other authors made the cross-cultural adaptation and validation of the OHIP-14 for Portuguese ^7^.

Differences in health are an expression of inequalities and inequities that require an ethical judgment as to what conditions are considered unacceptable. They reflect the disproportionate burden of diseases between segments of the population, including differences that occur due to sociodemographic characteristics, race or ethnicity, schooling level, and income. The disparities in oral health can be seen as unequal and unfair differences in oral health condition and access to dental care ^8^, such as the inequalities found between Indigenous and non-Indigenous people.

Although several researchers have documented the precariousness and deterioration of the oral health of Indigenous people, some authors have argued for a better investigation of this situation due to the profound socioeconomic and environmental changes Indigenous people have been undergoing - including their subsistence and food-related activities, factors that certainly alter oral health conditions ^9^ ^,^ ^10^. Furthermore, the consequences due to interethnic contact remain poorly explored ^9^ ^,^ ^10^. The scarce scientific literature describing the oral health profile of Brazilian Indigenous communities has focused on the prevalence of dental caries in restricted age groups of certain ethnic groups ^9^ ^,^ ^10^.

Subjective evaluations have become an essential critical contribution in finding vulnerable population groups, such as Indigenous communities. The literature has clearly established an association between negative self-perceived oral health and oral clinical conditions (oral diseases, tissue damage, pain, functional and aesthetic impairment) and unfavorable psychosocial, socioeconomic, demographic and behavioral factors as these groups need complex and often personalized interventions ^11^. In view of the scarce data in this area regarding the Indigenous population, this study aimed to evaluate the perception of the impact of oral problems on the quality of life of Indigenous residents in the municipality of Aracruz, Espírito Santo State, Brazil.

## Methods

This cross-sectional study was conducted to analyze the prevalence and incidence of chronic diseases in the Indigenous adult population in Aracruz.

The villages are located in the Aracruz Indigenous reserve, occupying an area of about 18,000 hectares on the northern coast of the state about 80km from the capital of state, Vitória. Its population spans an approximate contingent of 5,000 individuals. The reserve is divided into two demarcated areas, a larger one (about 15,000 hectares) that include five Guarani and six Tupiniquim villages. The other area of the reserve lies on the state coast (Reserva de Comboios). It has a single Tupiniquim village. 

The eligible population (≥ 20 years) consists of about 2,950 people, of which about 90% are Indigenous and the other 10% are non-Indigenous (usually people who have married Indigenous people and went to live in the chosen villages). After disseminating this project in the community, all those eligible were invited via personal contact with community health workers, of whom 1,084 were examined and interviewed in Vitória on a scheduled day.

Data were collected by questionnaires using the standardized personal interview method in a room capable of maintaining individuals’ privacy during a visit to the Cardiovascular Research Clinic of the Health Sciences Center at Federal University of Espírito Santo (UFES, acronym in Portuguese). On Tuesdays, Wednesdays, Thursdays, and Fridays during data collection, six participants traveled in a vehicle that attended the project to the clinic, arriving from 07:00 to 07:30, while fasting (for 10 to 14 hours) in order to perform tests and measurements. Examination lasted from September 2020 to July 2022.

Although familiar with their native languages, these Indigenous communities use Portuguese in their daily lives, speaking it fluently and having no difficulty understanding the questionnaires.

In this research sociodemographic data, schooling level, use of dental services in the last 12 months, and need for prostheses were chosen as independent variables.

OHIP score (considering the seven dimensions of impact on quality of life) was chosen as the dependent variable. A five-point Likert frequency scale was used to codify the answers. The answers “very often” and “fairly often” in the questionnaire were considered as “with impact on quality of life”, whereas the answers “occasionally”, “hardly ever”, or “never” were considered as “without impact”. As such, the results were dichotomized.

At the end of the interviews, participants’ oral cavity were visually and clinically examined by a dental surgeon and two undergraduate students of the dentistry course who had been trained to collect information about tooth loss. Examinations followed a standard procedure that respected individuals’ environmental conditions, physical location, diagnostic criteria, and data recording of the examination. Individuals seated facing the examiner, who used a wooden spatula and gauze to perform their functions. Since the data were collected during the COVID-19 pandemic, strict measures were taken to protect the examiners, including waterproof aprons, N95 respirators, hats, glasses, face shields, and disposable procedure gloves. Participants wore surgical masks during their interviews, only exposing their oral cavity for the minimal time necessary to observe their dental arches.

The differences between groups were evaluated using the chi-squared and Fischer’s exact tests for each independent variable and the seven OHIP dimensions. To evaluate the strength of the association between exposure and event, odds ratios (OR) were calculated with 95% confidence interval (95%CI). Thus, it was possible to know the frequency of impact per dimension. However, assessing the association between a predictor and the effect variable for all dimensions in tandem remain difficult. To solve this, the Mantel-Haenzsel method was used.

This research was funded by the Espírito Santo State Research Foundation and the Brazilian National Research Council (Pronex, notice 24/2018). This research project was approved by the Research Ethics Committee of the Health Sciences Center at UFES (opinion n. 3,655,623, approved on October 22, 2019) and the Brazilian National Research Ethics Commission (opinion n. 3,828,655, approved on February 9, 2020).

## Results

This study evaluated 1,084 participants, 37% of the population aged over 20 years. This sample had proportions similar to the demographic structure of the total population regarding age, ensuring a large sampling power for the prevalence of the impact of oral issues on quality of life (which is largely defined by age group).

Regarding sociodemographic data, we observed that most participants were women (57.7%), aged up to 30 years (27.5%), married (61.1%), with complete secondary education (36.9%). A total of 88.6% of participants declared themselves “Indigenous”, most of whom belonged to the Tupiniquim ethnicity (Table 1).

**Table 1:** Demographic data of the Indigenous people in this research.

Characteristics	n	%
Sex		
Male	459	42.3
Female	625	57.7
Age range (years)		
Up to 30	298	27.5
31-40	265	24.5
41-50	213	19.6
51-60	166	15.3
61 or older	142	13.1
Marital status		
Married/Living with spouse	662	61.1
Single	382	35.2
Widowed	37	3.4
Divorced	3	0.3
Schooling level		
Illiterate	55	5.1
Incomplete middle school	293	27.0
Complete middle school	166	15.3
Incomplete high school	57	5.3
Complete high school	400	36.9
Incomplete higher education	62	5.7
Complete higher education	51	4.7
Race		
Indigenous	972	88.6
White	37	3.4
Black	14	1.3
Mixed-race	57	5.3
Yellow	2	0.2
Other	2	0.2
Ethnicity		
Tupiniquim	864	79.7
Guarani	96	8.9
Other	13	1.2
Non-Indigenous	111	10.2
Total	1,084	100.0

The participants in this study also answered questions about use, such as most sought-after type of dental service. The most sought-after dental services referred to routine and preventive treatments (51.6%), followed by emergency treatments (11.6%). Regarding the impact on quality of life, we observed that Indigenous people who sought dental care for routine/prevention perceived a greater impact than those who used the service for urgent matters.

The most common type of dental service was that offered by the Brazilian Unified National Health System (SUS, acronym in Portuguese) (57.6%), followed by private services (30%).

The analysis of variable age groups found a statistical significance in functional limitation (OR = 3.322, 95%CI: 2.338-4.721), physical pain (OR = 2.020, 95%CI: 1.508-2.706), psychological discomfort (OR = 1.394, 95%CI: 1.033-1.881), physical disability (OR = 2.092, 95%CI: 1.441-3.036), psychological disability (OR = 1.543, 95%CI: 1.119-2.128), and handicap (OR = 2.156, 95%CI: 1.450-3.205). In the Mantel-Haenszel test (OR = 1.983, 95%CI: 1.517-2.591), the impact was approximately 1.9 times greater for individuals up to 50 years of age, while the functional limitation dimension had a 3.3 times greater impact in individuals aged 51 or older (OR = 3.322, 95%CI: 2.338-2.706) (Table 2).

**Table 2:** Impact caused by oral issues according to the age group of the Indigenous people in this research.

Dimension	Up to 50 years		51 years or older		Significance	OR (95%CI)
	n	%	n	%		
Functional limitation					0.000	3.322 (2.338-4.721)
With impact	73	48.0	79	52.0		
No impact	703	75.4	229	24.6		
Physical pain					0.000	2.020 (1.508-2.706)
With impact	160	60.2	106	39.8		
No impact	616	75.3	202	24.7		
Psychological discomfort					0.019	1.394 (1.033-1.881)
With impact	173	66.3	88	33.7		
No impact	603	73.3	220	26.7		
Physical disability					0.000	2.092 (1.441-3.036)
With impact	76	57.1	57	42.9		
No impact	700	73.6	251	26.4		
Psychological disability					0.006	1.543 (1.119-2.128)
With impact	132	64.1	74	35.9		
No impact	644	73.3	234	26.7		
Social disability					0.058	1.841 (0.936-3.619)
With impact	21	58.3	15	41.7		
No impact	755	72.0	293	28.0		
Handicap					0.000	2.156 (1.450-3.205)
With impact	64	56.1	50	43.9		
No impact	712	73.4	258	26.6		
Mantel-Haenszel					0.000	1.983 (1.517-2.591)

95%CI: 95% confidence interval; OR: odds ratio.

The results showed that the following dimensions had significant associations with a need for removable total dentures: functional limitation (OR = 4.798, 95%CI: 3.237-7.113), physical pain (OR = 2.437, 95%CI: 1.699-3.496), physical disability (OR = 2.125, 95%CI: 1.361-3.318), and handicap (OR = 2.016, 95%CI: 1.252-3.246). In the Mantel-Haenszel test (OR = 2.384, 95%CI: 1.667-3.409), the impact was 2.3 times greater for people who did not require removable (Table 3).

**3:** Impact caused by oral issues according to the need for removable total dentures in the Indigenous participants in this research.

Dimension	Requires dentures		Does not require dentures		Significance	OR (95%CI)
	n	%	n	%		
Functional limitation					0.000	4.798 (3.237-7.113)
With impact	54	35.5	98	64.5		
No impact	96	10.3	836	89.7		
Physical pain					0.000	2.437 (1.699-3.496)
With impact	61	22.9	205	77.1		
No impact	89	10.9	729	89.1		
Psychological discomfort					0.463	1.038 (0.695-1.549)
With impact	37	14.2	224	85.8		
No impact	113	13.7	710	86.3		
Physical disability					0.001	2.125 (1.361-3.318)
With impact	31	23.3	102	76.7		
No impact	119	12.5	832	87.5		
Psychological disability					0.406	1.077 (0.699-1.659)
With impact	30	14.6	176	85.4		
No impact	120	13.7	758	86.3		
Social disability					0.050	2.144 (0.988-4.654)
With impact	9	25.0	27	75.0		
No impact	141	13.5	907	86.5		
Handicap					0.004	2.016 (1.252-3.246)
With impact	26	22.8	88	77.2		
No impact	124	12.8	846	87.2		
Mantel-Haenszel					0.000	2.384 (1.667-3.409)

95%CI: 95% confidence interval; OR: odds ratio.

The analysis also showed that a need for removable partial dentures impacted all OHIP dimensions: functional limitation (OR = 3.555, 95%CI: 2.483-5.089), psychological discomfort (OR = 2.190, 95%CI: 1.650-2.905), physical disability (OR = 2.517, 95%CI: 1.740-3.640), psychological disability (OR = 2.857, 95%CI: 2.093-3.899), social disability (OR = 2.343, 95%CI: 1.194-4.598), and handicap (OR = 2.908, 95%CI: 1.951-4.336). In the Mantel-Haenszel test (OR = 2.538, 95%CI: 1.974-3.264), the impact on quality of life was 2.5 times greater for individuals requiring removable partial dentures (Table 4). The exception referred to physical pain (OR = 1.735, 95%CI: 1.311-2.296), which more greatly impacted individuals who had no need for removable partial dentures.

The prevalence of overall impact equaled 45.7%, as 495 Indigenous individuals reported an impact of oral issues on their quality of life, whereas physical pain (24.5%) and psychological discomfort (24.1%) had the highest impact percentages (Table 5).

**Table 4:** Impact caused by oral issues according to the need for removable partial dentures in the Indigenous participants in this research.

Dimension	Requires dentures		Does not require dentures		Significance	OR (95%CI)
	n	%	n	%		
Functional limitation					0.000	3.555 (2.483-5.089)
With impact	98	64.5	54	35.5		
No impact	315	33.8	617	66.2		
Physical pain					0.000	1.735 (1.311-2.296)
With impact	128	48.1	138	51.9		
No impact	285	34.8	533	65.2		
Psychological discomfort					0.000	2.190 (1.650-2.905)
With impact	137	52.5	124	47.5		
No impact	276	33.5	547	66.5		
Physical disability					0.000	2.517 (1.740-3.640)
With impact	77	57.9	56	42.1		
No impact	336	35.3	615	64.7		
Psychological disability					0.000	2.857 (2.093-3.899)
With impact	121	58.7	85	41.3		
No impact	292	33.3	586	66.7		
Social disability					0.010	2.343 (1.194-4.598)
With impact	21	58.3	15	41.7		
No impact	392	37.4	656	62.6		
Handicap					0.000	2.908 (1.951-4.336)
With impact	70	61.4	44	38.6		
No impact	343	35.4	627	64.6		
Mantel-Haenszel					0.000	2.538 (1.974-3.264)

95%CI: 95% confidence interval; OR: odds ratio.

**Table 5:** General impact caused by oral issues in the Indigenous participants of this research.

Dimension	With impact		No impact	
	n	%	n	%
Physical pain	266	24.5	818	75.5
Psychological discomfort	261	24.1	823	75.9
Psychological disability	206	19.0	878	81.0
Functional limitation	152	14.0	932	86.0
Physical disability	133	12.3	951	87.7
Handicap	114	10.5	970	89.5
Social disability	36	3.3	1048	96.7
Total	495	45.7	589	54.3

## Discussion

Socio-dental indicators, which are based on self-perception and the impacts brought by oral issues, offer important advantages for planning and organizing dental services, highlighting the paradigm shift from an exclusive emphasis on purely biological to psychological and social aspects ^12^. This took place to better understand patients’ fears and desires so dental care occurs in an individualized and comprehensive manner, promoting patients’ health and well-being. Applying subjective indicators has brought great advances to epidemiological studies in Dentistry ^13^.

This novel approach has been very useful in health planning ^14^. Contemporary Indigenous communities, although on a different time scale and under other human, social, economic, and environmental factors, have experienced socioeconomic and ecological changes when in contact with other societies, which can greatly change oral health conditions. The literature documents deteriorating oral health conditions in Indigenous people ^15^ ^,^ ^16^, which this research also found - according to its 45.7% prevalence of impact of oral issues on Indigenous people’s quality of life, whereas physical pain (24.5%) and psychological discomfort (24.1%) showed the highest number of individuals with impacts.

Some studies in non-Indigenous populations have found lower prevalences of the impact of oral problems on quality of life, such as the prevalence of 35% in a population-based study conducted in the municipality of Marechal Floriano (Espírito Santo State) ^13^, 29% in a survey with community health workers in Vitória ^17^, and 32.6% in a study conducted in care centers for older adults in Vitória ^18^.

A survey with workers of a mixed economy company in the municipality of Vitória, found a 7.8% prevalence of impact, much lower than that normally found in studies with non-Indigenous populations. The authors argue that this low prevalence of impact can be explained due to the higher schooling and income in their sample ^19^.

This high prevalence of the impact on quality of life shows the need for adequate oral health planning that meets the specificities of Indigenous populations due to their specific characteristics. In addition to having abandoned traditional cultivation practices (which in the past contributed to a greater variety of available food), Indigenous communities have undergone socioeconomic transformations, which places them under great vulnerability in the face of food- and oral health-related issues ^20^.

The solution to the high prevalence of impacts of oral issue related to quality of life is certainly linked to access to comprehensive dental treatment. This includes specialized treatment for those with high prevalence of clinical and perceived needs - especially for groups under unfavorable socioeconomic contexts, for which the cost of treatment is the main barrier ^21^. This study also evinced such aspect as most participants (57.6%) sought the dental care service offered by SUS, a public and free service.

Individuals who sought dental care for routine/preventive reasons reported a greater impact on their quality of life, a result unlike that found by studies conducted in non-Indigenous populations ^13^ ^,^ ^17^ ^,^ ^19^ ^,^ ^22^.

The territory of these Indigenous communities include three health units that have dental offices: Pau Brazil, Caieiras Velha, Comboios. Moreover, a dental surgeon works at the health units of Boa Esperança and Irajá. Therefore, the coverage of dental service by SUS is almost 100%, which certainly facilitated the use of the service for routine/prevention. However, the greater impact for these individuals may stem from infrastructure problems with the equipment and the lack of reference for specialized dental treatments. Thus, individuals had access to the service but did not have their situations resolved.

Considering the results of this research according to age groups, individuals aged up to 50 years had greater impact in almost all dimensions, except for functional limitation, in which individuals aged 51 years or more had greater impact. This result differs from those in other studies conducted in Brazil ^23^ ^,^ ^24^, which found greater impact in those aged over 40 years. However, in addition to the difference in cut-off point of the age group, which makes it difficult to compare results, Indigenous and non-Indigenous populations show different socioeconomic and cultural differences that can explain such result.

The evaluation of need for dentures showed a greater impact in those who required removable partial dentures, a result often found in other studies with non-Indigenous people ^13^ ^,^ ^17^ ^,^ ^18^ ^,^ ^19^ ^,^ ^25^. This is possibly explained by the prioritization of Brazilian public policies directed to children.

Until recently, the State only guaranteed adults access dental extractions, (which are mutilative in nature). The 2003 *Brazilian National Household Sample Survey* (PNAD, acronym in Portuguese) ^26^ evinced such exclusion, showing that the system excluded more than 27 million people. The results of the 2023 *Brazilian National Oral Health Survey* (SB Brasil, acronym in Portuguese) ^27^ showed similar results, evincing that 63.1% of those aged from 65 to 74 years used total dentures. A subjective indicator can demonstrate this accumulated need for treatment. In developed countries, the impact produced by the need for dentures is not measured, suggesting the need from greater attention to this age group ^23^.

In 2004, the Brazilian National Oral Health Policy (*Brasil Sorridente* - Smiling Brazil) proposed the reorganization of oral healthcare. Its strategic points include reducing social inequalities in oral health, expanding the coverage of oral health teams in the Brazilian Family Health Strategy (FHS), and stimulating the reorientation of the care model by improvements in the work of the oral health teams and oral health surveillance by increasing coverage through the centers of dental specialties (CEO, acronym in Portuguese) ^28^.

However, the oral healthcare currently offered by SUS is unable to meet the great repressed demand in the overall Brazilian population, as per the results of the SB Brasil 2023 ^27^ including adults aged 65 to 74 years, in which 49.58% reported need for dentures. This stands out even more in vulnerable populations such as Indigenous people, who already experience greater precariousness in oral health conditions due to changes in their eating habits, socioeconomic and environmental conditions, and due to the lack of preventive programs for dental diseases ^20^ and public policies that meet the particularities of these people.

The results regarding the need for removable total dentures showed a greater impact on Indigenous people who had no need for it; unlike the data found in research conducted with non-Indigenous individuals ^13^ ^,^ ^17^ ^,^ ^25^. This may be explained by the small number of individuals who reported needing removable total dentures (13.8%) and the social and cultural differences of Indigenous people.

The results of this research show the difficulties of these Indigenous communities regarding oral issues and the need for integrated and organized health planning with resolutive actions to meet the demands of these communities with specific social, cultural, and economic characteristics. Healthcare, a right of everyone and a duty of the State, should reach the entire population regardless of their geographical location or socioeconomic and cultural condition. This research highlights the applicability and strategies for its effective promotion and fulfilment. Brazil is remarkably diverse in its people and regions, yet there is a lack of health services for its native Indigenous population ^29^.

The inequalities in access to dental care services clearly show the vulnerability of Indigenous peoples. Interventions without planning that considers universal access and equity generate no benefits beyond widening health inequalities ^10^. Moreover, a lack of knowledge about cultural practices can prevent healthcare providers from understanding the methods these peoples use to treat diseases by other rationales or traditional medicinal treatments. The deep-rooted presence of the biomedical model means that the distance between healthcare providers and service users results in a lack of knowledge about the real problems of these communities ^10^.

As Indigenous people have an ancestral cultural background passed down from generations, dentists must provide more thoughtful care, including mutual approximation and exchange of knowledge, so they can learn about the living conditions in the Indigenous territory, oral health habits, and patients’ concept of their oral health, hygiene habits, and diet ^30^.

Thus, dental surgeons should acquire a cultural competence that transcends the limits of a welfare model focused on the individual, which are characteristics of the old medical model that remain well-established. This scenario would lead to prevention with a view to collective issues, respecting and valuing the social and cultural aspects of Indigenous people ^31^ and developing specific and qualified health promotion actions, characteristics of the new biopsychosocial model that meet the real needs in oral health of these people, thus improving their quality of life.

Researchers have raised concerns about the ability of psychometric instruments to measure constructs that capture dimensions of health with distinct health concepts ^32^. However, the literature has few studies that aimed to adapt health-related quality of life instruments that consider the unique way in which Indigenous cultures view their health and well-being. Differences in the structures of the OHIP-14 dimensions and Indigenous communities may stem from their experience of colonization, trauma, and racism and their deep connection to land, spirituality, and community ^33^.

A limitation of this study is its application of the OHIP-14 in a different culture, which may have caused differences in the extent of the attributes measured due to the distinction between the concepts of health and disease between cultures. However, this research represents the first study with a quality-of-life indicator in these communities and many others as this study found no studies using OHIP-14 in Brazilian Indigenous populations. Therefore, this unprecedented study in this region can serve as a basis for an initial assessment of the impact of oral health conditions on these peoples and as a resource for future research on this topic.

Moreover, the short Brazilian version of the OHIP-14 has good psychometric characteristics, representing a valuable tool for international research as it remains valid and reliable across cultures ^34^. The cross-sectional design of this research can also be considered a limitation, as it is not possible to establish a cause/effect relationship. However, its current, original, and highly relevant topic increases its importance despite its cross-sectional design.

Based on the analysis of this study results, we conclude that the prevalence of impact of oral issues on the quality of life in Indigenous villages in the municipality of Aracruz totaled 45.7%, higher than the impact for non-Indigenous individuals. Such a high percentage is an alert for health authorities, showing the vulnerable oral health of this population. Oral issues often negatively impact quality of life and widen the inequalities between Indigenous and non-Indigenous regarding planning and public policies in oral health.

Indigenous people aged up to 50 years of age who used the dental service for routine/preventive reasons, with a declared need for removable partial dentures and no need for removable total dentures reported a greater impact of oral issues on their quality of life.

## Data Availability

The research data are available upon request to the corresponding author.
